# Pilot study on agricultural pesticide poisoning in Burkina Faso

**DOI:** 10.2478/intox-2013-0027

**Published:** 2013-12

**Authors:** Adama M. Toe, Mustapha Ouedraogo, Richard Ouedraogo, Sylvain Ilboudo, Pierre I. Guissou

**Affiliations:** 1 Research Institute of Health Sciences, CNRST, Burkina Faso; 2 Laboratory of Toxicology and Pharmacology, Health Sciences Faculty, University of Ouagadougou, Burkina Faso

**Keywords:** pesticides, poisoning, farmers, Burkina Faso

## Abstract

Epidemiologic data related to agricultural pesticide poisoning cases in Burkina Faso were collected. The study was carried out using retrospective (from January 2002 to June 2010) surveys conducted among farmers and healthcare centers. One hundred and fifty-three (153) pest control products were recorded during the survey and 56 active ingredients were identified. Out of the 153 pest control products, 49 (*i.e.* 32%) were authorized for sale in Burkina Faso. The main risk factors are socio-demographic characteristics of farmers, their low education level, and some attitudes and practices on using agricultural pesticides. Pesticide poisonings are relatively frequent and their management was not always efficacious. Actions are needed to reduce pesticide poisoning as a global public health problem and to improve management of pesticide poisoning. To this purpose, advanced investigations should be carried out over a longer period of time to complement the present pilot study.

## Introduction

The agricultural sector is very important in the national economy of Burkina Faso. As a matter of fact, it employs 86% of the total population and generates about 40% of the gross domestic product (GDP). Diseases and animal pests cause major damage in agriculture and can be responsible in some cases for up to 30% of yield losses in Burkina Faso. Thus plant protection products are used to eradicate pests affecting crops, particularly in the case of intensive cultures such as cash crops, sugarcane, vegetable crops, and to a lesser extent fruit trees (MAHRH, 2007). In 1997, more than 2 500 tons of pesticide formulations were estimated to be used in Burkina Faso and that only for the treatment of cotton, vegetables and the consumption of plant protection services (Van Der Valk & Diarra, 2000). The annual growth rate of pesticide consumption reached 11% (Toe & Kinane, 2004). Pesticides are considered as one of the main factors of rural development at a time when demographic and economic constraints increase the pressure for productivity growth. They help to reduce the damage caused to crops by pests and even to prevent them. However, pesticides constitute a real threat for health and environment in Burkina Faso (Ouédraogo *et al.*, 2009).

Several studies carried out in Burkina Faso have shown that agricultural producers did not follow good agricultural practices (Domo, 1996; Ouédraogo *et al.*, 2009; Toe *et al.*, 2002; Toe *et al.*, 2012). Yet, to the best of our knowledge, recent data on agricultural pesticide poisoning in Burkina Faso are not available. Our study aimed at collecting epidemiologic data related to agricultural pesticide poisoning cases in Burkina Faso.

## Methods

### Study area

**Figure 1:**
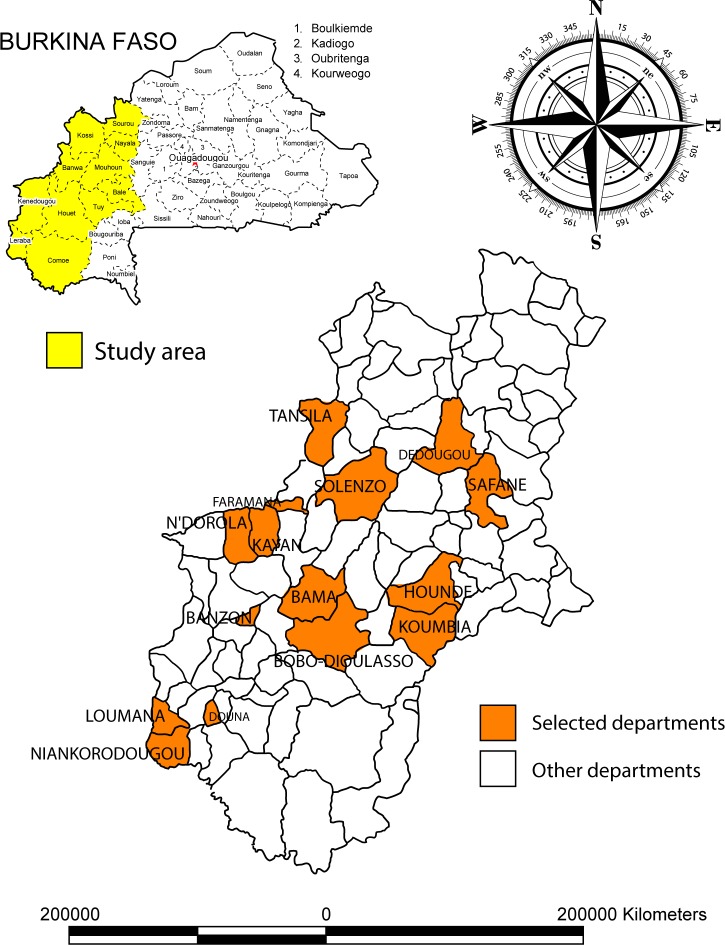
Departments hosting survey sites.

Field work (surveys and interviews) took place in the agricultural areas of the “Hauts-Bassins”, the “Cascades” and the “Boucle du Mouhoun”. They are the biggest cotton producing zones of Burkina Faso and the major users of agricultural pesticides. The “Hauts-Bassins”, the “Boucle du Mouhoun” and the “Cascades” regions had a population of 1 389 258 inhabitants, 1 478 392 inhabitants, and 430 677 inhabitants, respectively in 2006, *i.e.* about 23% of the national population. Survey sites were selected on the basis of their agro-climatic characteristics, their geographic situation, the extent of cultivated crops such as cotton, maize and rice on which pesticides were highly used. The sites were selected on the basis of the above-mentioned criteria (Figure 1).

### Design of the study

Relevant administrative and technical services were contacted to collect preliminary data on the number of farms and their different categories. On the basis of the data obtained, a random sampling was done to identify persons to be surveyed.

Prospective studies were conducted to monitor agricultural producers during pesticide application operations and to identify weaknesses and strengths of producers’ pesticide management (type of pesticide, safety measures, management of agro-chemical stocks, left-over pesticides).

As for epidemiological data from pesticide-related poisoning, a retrospective study was done. It was conducted from June to July, 2010. All pesticide-related poisoning cases admitted in healthcare centers from January 2002 to June 2010 were included.

In each department (survey site), farmers of fifty farms were selected. In order to take into consideration the different categories of agricultural producers, a stratified sampling based on the size of the farms was created. Based on the size of farms, the following four groups were taken into account:

Group I: Less than 1000 m^2^

Group II: Between 1000 and 2500 m^2^

Group III: Between 2500 and 5000 m^2^

Group IV: More than 5000 m^2^

The total number of farms per department and the number of farms of each group was assessed in order to do the sampling. The representativeness (group coefficient) of each group in the department was calculated on the basis of the total number of farms per group as follows:

$$\frac{\text{Number of farms in the group}}{\text{Total number of farms in the department}}$$

To determine the number of farms from each group that should be part of the fifty farms selected for the sampling, we multiplied 50 by the group coefficient.

All the healthcare centers of the survey sites were systemically included to the study.

### Investigations among farmers and healthcare centers

Investigations among farmers consisted in collecting data on pesticides used by farmers and their attitude when poisoning by pesticides would occur. In healthcare centers, surveys aimed to record poisoning incidents. The investigations were designed to collect reliable and well-documented information. Following a questionnaire, interviews were conducted among healthcare agents to record and describe poisoning incidents caused by pesticides.

### Data processing and analysis

After the perusal of survey sheets, data were codified, entered and analyzed using the data management software Epi Info 3.3.2 and Excel 2007 software. Results were summarized into descriptive statistics.

## Results

### Risk factors of poisoning

A total of 650 farmers distributed in 16 villages of the three regions studied were surveyed. Pesticides were mostly handled by men. In fact, 98.3% of the surveyed persons involved in the application of pesticides were men. The average age of the farmers was 39.58±10.30 years. The youngest person involved in pesticide application operations was 17 years old and the oldest one was 75; 15.3% of the farmers were more than 50 years old.

One hundred and fifty-three (153) pest control products (pesticides) were recorded during the survey and 56 active ingredients were identified (Table 1). Out of the 153 pest control products, 49 (*i.e.* 32%) were authorized for sale by the Sahelian Pesticide Committee, hence in Burkina Faso. Pesticides of classes I_b_, II, III and IV (WHO classification) were indistinctly used. The main categories of pesticides found were herbicides, insecticides and fungicides. The majority of the surveyed population (60.5%) had no education at all, 31.8% of them had primary education, and 7.7% had a secondary education level. Thirty-nine percent of the farmers had less than 10 years’ experience in pesticide use, whereas 54% had between 10 and 30 years’ experience.

**Table 1:** Pesticide formulations which were identified during the survey among dealers.

Formulation	Active ingredients	Pesticide category	WHO Class	Sources of chemicals
ACEPRONET 400	Acetochlore	Herbicide	III	China
	Prometryne			
ACTELLIC SUPER	Pyrimiphos-methyl	Insecticide		France
	Permethrine			
ACTELLIC 50	Pyrimiphos-methyl	Insecticide	III	Switzerland
ACTELLIC SUPER	Pyrimiphos-methyl	Insecticide		SAPHYTO
	Permethrine			
ACTION 80 DF	Diuron	Herbicide		SCAB
ADWUMA WURA	Glyphosate	Herbicide		China
ADWUMA WURA 75.7%	Glyphosate	Herbicide		China
ADWUMAMU HENE	Glyphosate	Herbicide		
AGRAZINE 500	Atrazine	Herbicide		China
AGRAZINE 80 WP	Atrazine	Herbicide		France/China
AGRAZINE 90	Atrazine	Herbicide		China/France
AGRAZINE DF	Atrazine	Herbicide		France
AKIZON 40 SC	Nicosulfuron	Herbicide	III	France
ALLIGATOR 400 EC	Pendimethaline	Herbicide	III	France
APRON PLUS 50 DS	Metalaxyl-M	Insecticide		
	Carboxine			
	Furathiocarbe			
APRON STAR 42 WS	Thiamethoxam	Insecticide		Switzerland
	Metalaxyl-M			
	Difenoconazole			
ATRAHERB	Atrazine	Herbicide		China
ATRALM 500	Atrazine	Herbicide		SENEFURA/SCAB
ATRALM 90	Atrazine	Herbicide		SENEFURA
ATRAVIC 500 SC	Atrazine	Herbicide		SAPHYTO
ATRAZ 50	Atrazine	Herbicide		Cantonments Accra
ATRAZ 80 WP	Atrazine	Herbicide		SARO AGROCHEM
ATRAZILA 500	Atrazine	Herbicide		Kumark Trading Ent.
ATRAZILA 80 WP	Atrazine	Herbicide		Shenzhen Baocheng Chemical industry co. Ltd
ATRAZINE	Atrazine	Herbicide		Japan
ATRAZINE WEEDICIDE	Atrazine	Herbicide		Japan
AVAUNT 150 EC	Indoxacarb	Insecticide	II	SOFITEX/SAPHYTO
BACCARA 335 EC	Propanil	Herbicide		SAPHYTO
	2,4 D			
BENAXONE SUPER	Paraquat	Herbicide		Bentronic Productions
BEXTRA	2,4 D	Herbicide		CalliGhana/Ghana Bentronic Production
BISTAR 10 WP	Bifenthrine	Insecticide	II	
BLAST 46 EC	Lambdacyhalothrine	Insecticide		SAPHYTO
	Acetamipride			
CAIMAN ROUGE	Endosulfan	Insecticide	II	SOFITEX/SSI
	Thirame			
CAIMAN SUPER	Alphacypermethrine	Insecticide		SSI
	Endosulfan			
CALFOS 500 EC	Profenofos	Insecticide	II	SAPHYTO
CALLIFOR	Prometryne	Herbicide		SAPHYTO
	Fluometuron			
CALLIFOR 500	Prometryne	Herbicide	III	SAPHYTO
	Fluometuron			
CALLIFOR G	Prometryne	Herbicide	III	SAPHYTO
	Fluometuron			
	Glyphosate			
CALLIHERB	2,4 D of amine salt	Herbicide		SAPHYTO
CALLIMAN 80 WP	Manebe	Fongicide		Callivoire
CALLITRAZ 90 WG	Atrazine	Herbicide		SAPHYTO
CALLOXONE SUPER	Paraquat	Insecticide		SAPHYTO
CALRIZ	Propanil	Herbicide		SAPHYTO
	Trichlopyr			
CALTHIO C	Chlorpyrifos-ethyl	Insecticide		SAPHYTO/FASOCOTON
	Thirame			
CALTHIO DS	Lindane	Insecticide		SAPHYTO
	Thirame			
CALTHIO E	Endosulfan	Insecticide		SCAB
	Thirame			
CAPT 80 EC	Acetamipride	Insecticide		SAPHYTO
	Cypermethrine			
CAPT 88 EC	Acetamipride	Insecticide	II	Ivory Coast /ALM
	Cypermethrine			
CARBODAN 3% G	Carbofuran	Insecticide		Makhteshim Agan France
CELTACAL 12,5 EC	Deltamethrine	Insecticide		SAPHYTO
CIGOGNE	Profenofos	Insecticide		STEPC Abidjan
	Cypermethrine			
CODAL gold 412,5 DC	S-Metolachlore	Herbicide	III	SAPHYTO/SYNGENTA
	Prometryne			
CONQUEST C 88 EC	Cypermethrine	Insecticide	II	SAPHYTO
	Acetamipride			
CONQUEST C 176 EC	Acetamipride	Insecticide	II	SAPHYTO
	Cypermethrine			
COTODON PLUS 500 EC	Metolachlore	Herbicide	III	NOVARTIS
	Atrazine			
COTONET 500 EC	Metolachlore	Herbicide		DTE SA Chine
	Terbutryne			
CURACRON 500 EC	Profenofos	Insecticide	III	SOFITEX
CYPERCAL 25 EC	Cypermethrine	Insecticide		SAPHYTO
CYPERCAL 50 EC	Cypermethrine	Insecticide	III	SAPHYTO
CYPERCAL P 690 EC	Profenofos	Insecticide	II	SAPHYTO
	Cypermethrine			
CYPERPHOS	Cypermethrine	Insecticide		Bayer crop science
	Triazophos			Bayer crop science
CYRENS 480 EC	Chlorpyrifos-ethyl	Insecticide		SAVANA
DECIS	Deltamethrine	Insecticide		STEPC/Bayer crop science
DECTACOL 12,5	Deltamethrine	Insecticide		SAPHYTO
DIAFURAN	Carbofuran	Insecticide		SAPHYTO
DIGA FAGALAN 360 SL	Glyphosate	Herbicide	III	PROPHYMA/SAVANA
DIURALM 80 WG	Diuron	Herbicide	III	SENEFURA/ALM
DOMINEX 100	Alpha cypermethrine	Insecticide		
DUREXA	Chlorpyrifos-ethyl	Insecticide		SAPHYTO
ENDOCOTON 500 EC	Endosulfan	Insecticide	Ib	SAPHYTO
FANGA 500 EC	Profenofos	Insecticide	II	SENEFURA
FOCUS GLYPHOSATE 360 SL	Glyphosate	Herbicide		SOFITEX
FOCUS Ultra 100 EC	Cycloxydime	Herbicide	III	BASF/Tech Agro International
FURADAN 5G	Carbofuran	Insecticide		SCAB/FMC
FUSILADE	Fluazifop-p-butyl	Herbicide	III	SCAB
GALAXY 450 EC	Clomazone	Herbicide		SENEFURA/SAPHYTO
	Pendimethaline			
GALLANT SUPER	Haloxyfop-R-methyl	Herbicide	III	Callivoire
GARIL 432 EC	Trichlopyr	Herbicide	II	SAPHYTO
	Propanil			
GLYCEL 410 SL	Glyphosate	Herbicide	II	Top phyt/ Topex Agro Elevage Developpement SARL CONAKRY
GLYPHADER	Glyphosate	Herbicide		SCAB
GLYPHADER 480	Glyphosate	Herbicide		Golden stork
GLYPHADER 75	Glyphosate	Herbicide	III	SCAB
GLYPHALM 500 WG	Glyphosate	Herbicide	III	SENEFURA/ALM
GLYPHALM 360 SL	Glyphosate	Herbicide	III	SENEFURA/ALM
GLYPHALM 720	Glyphosate	Herbicide		SENEFURA
GLYPHONET 360 SL	Glyphosate	Herbicide	III	DTE SA Chine
GLYSATE	Glyphosate	Herbicide		Yaw wussma Ventures
GRAMOQUAT SUPER	Paraquat chloride	Insecticide		Kumark Trading Ent.
GRAMOXONE SUPER	Paraquat	Insecticide	II	SCAB
HALONET SUPER 104 EC	Haloxyfop-R-methyl	Herbicide	III	DTE SA Chine
HERBALM	2,4 D of amine salt	Herbicide		SENEFURA/ALM International
HERBEXTRA 720 SL	2,4 D of amine salt	Herbicide	II	SCAB, Kumark Trading Ent., SSI
HERBEXTRA 750 SL	2,4 D of amine salt	Herbicide		SCAB
HERBISUPER	Acetochlore	Herbicide	II	SCAB
	Atrazine			
HERBIMAIS	Atrazine	Herbicide		SCAB
	Nicosulfuron			
IBIS A	Alphacypermethrine	Insecticide		SCAB/SSI
	Acetamipride			
IBIS P	Alphacypermethrine	Insecticide		SSI
	Profenofos			
IKOKADIGNE	Haloxyfop-R-methyl	Herbicide	II	SCAB
KALACH 360 SL	Glyphosate	Herbicide	III	SAPHYTO/CalliGhana
KALACH EXTRA 70 SG	Glyphosate	Herbicide	III	SAPHYTO
KAMAXONE	Paraquat	Insecticide		Kumasi/Ghana
KART 500 SP	Cartap	Insecticide	II	STEPC
KOMBAT	Lambdacyhalothrine	Insecticide		SARO
KUAPA WARA	Glyphosate	Herbicide		
KUM NWURA	Glyphosate	Herbicide		
LAGON 380 SC	Isoxaflutol	Herbicide	III	STEPC/Bayer crop science
	Aclonifene			
LAMBDA SUPER	Lambdacyhalothrine	Insecticide		SCAB, Kumark Trading Ent.
LAMBDACAL P 212 EC	Profenofos	Insecticide	II	SAPHYTO
	Lambdacyhalothrine			
LAMBDACAL P 636 EC	Profenofos	Insecticide	II	SOFITEX
	Lambdacyhalothrine			
LAMDEX 430 EC	Lambdacyhalothrine	Insecticide	II	Makhteshim Chemical Works
	Chlorpyrifos-ethyl			
LASSO	Atrazine	Herbicide	III	SCAB/Candel
	Alachlore			
MALIK 108 EC	Haloxyfop-R-methyl	Herbicide	III	SAVANA
MALO BINFAGA	2,4 D	Herbicide	II	SAVANA
MILSATE	Glyphosate	Herbicide		Topaz Multi industrie Ghana
MITOX	Fenvalerate	Insecticide		Bentronic Productions
MOMTAZ 45 WS	Imidaclopride	Insecticide	III	PROPHYMA/SAVANA
	Thirame			
NICOMAIS 40	Nicosulfuron	Herbicide	III	PROPHYMA/SAVANA
NWURA WURA	Glyphosate	Herbicide		
OXARIZ 250 EC	Oxadiazon	Herbicide	III	SAVANA
PACHA 25 EC	Lambdacyhalothrine	Insecticide	II	SAVANA
	Acetamipride			
PHOSTOXIN	Phosphure d'alumine	Insecticide		Kumark Trading Ent.
POWER	Glyphosate	Herbicide		
POWER GLYPHOSATE 480I._P.A	Glyphosate	Herbicide		
PRIMAGRAM 360	Atrazine	Herbicide		SYNGENTA
	S-Metalochlore			
PROTECTOR	Lambdacyhalothrine	Insecticide		SENEFURA, SOFITEX/AF-Chem SOFACO-CI
	Pyriproxyfene			
RISTAR	Oxadiazon	Herbicide		SCAB
RIZTOP 250 EC	Oxadiazon	Herbicide		SAPHYTO
ROCKY 386 EC	Endosulfan	Insecticide	III	SAPHYTO
	Cypermethrine			
RONSTAR PL	Oxadiazon	Herbicide		SAPHYTO/Bayer crop science
	Propanil			
ROUNDUP 360 SL	Glyphosate	Herbicide	III	SCAB
ROUNDUP 680	Glyphosate	Herbicide		SCAB
ROUNDUP 680 BIOSEC	Glyphosate	Herbicide		SCAB
ROUNDUP TURBO	Glyphosate	Herbicide	III	SCAB
SAMORY	Bensulfuron-methyl	Herbicide	III	SCAB
SELECT 120 EC	Clethodim	Herbicide	III	SAPHYTO
SHARP	Glyphosate	Herbicide		Kumark Trading Ent.
SHARP 80 g/L	Glyphosate	Herbicide		
SHYE NWURA	Glyphosate	Herbicide		
SINOSATE	Glyphosate	Herbicide		Natosh Enterprise AGRO-DIVISION Ghana
STOMP	Pendimethaline	Herbicide		SENEFURA/BASF
STOMP 500 EC	Pendimethaline	Herbicide		SOFITEX
SUPRAXONE	Paraquat	Insecticide		Golden stork
TARGA SUPER 50	Quizalofop-p-éthyl	Herbicide		SAPHYTO/SOFITEX
TEMPRA	Diuron	Herbicide		SAPHYTO
TERMICAL 480 EC	Chlorpyrifos-ethyl	Insecticide		SAPHYTO
TIHAN 175 O-TEQ	Spirotetramate	Insecticide	III	SCAB/Bayer crop science
	Flubendiamide			
TITAN 25 EC	Acetamipride	Insecticide		SAPHYTO
TOPSTAR	Oxadiargyl	Herbicide	III	SCAB, SAPHYTO
TOUCHDOWN	Glyphosate	Herbicide		SYNGENTA
TOUCHDOWN HI TECH	Glyphosate	Herbicide		
TRAZINE	Atrazine	Herbicide		Bentronic Productions
WEED FAST	Glyphosate	Herbicide		WEYOUNG CW Kumassi

Our study showed that the pesticide application equipment used was mainly backpack sprayers with a volume capacity of 10 to 20 liters (in 96% of cases) and Ultra Low Volume sprayers (ULV) or Ultra Bas Volume (UBV) sprayers with a volume capacity ranging from 1 to 5 liters (4% of cases).

Some of the farmers (24.45%) reported not having any left-over pesticides as they knew the exact quantities required for treatment. Most of the surveyed farmers (69.12%) kept their unused pesticides for further applications. They stored them at their place or in the fields. A few of them declared dumping them into nature (4.86%) or burying them (1.72%).

**Figure 2:**
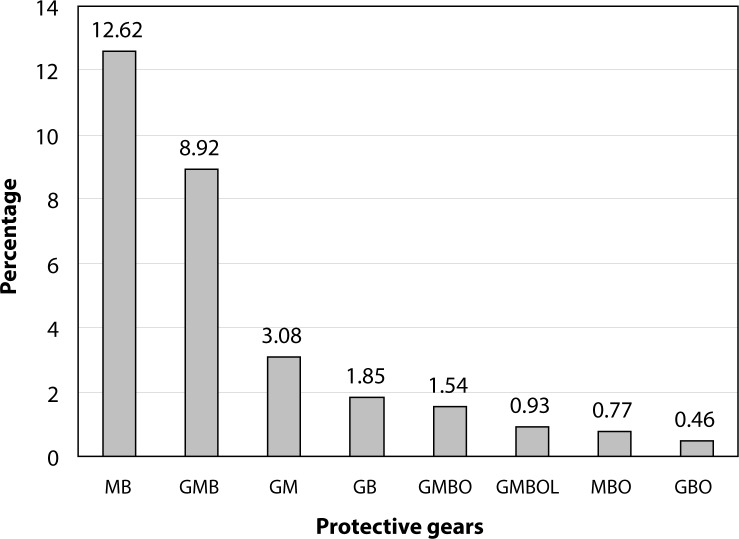
Percentage of farmers (involved in the application of pesticides) wearing combination of protective gears. MB: masks + boots; GMB: gloves + masks + boots; GM: gloves + masks; GB: gloves + boots; GMBO: gloves + masks + boots + overall; GMBOG: gloves + masks + boots + overall + glasses; MBO: mask + boots + overall; GBO: gloves + boots + overall.

The individual protective equipments that were widely used by farmers were masks (40% of farmers use them) followed by boots (28.8%), while overalls tend to be seldom used (4.5%). Only rarely did the farmers use a combination of two or more protective gears (Figure 2). Very few farmers have full protection (0.93%).

The majority of the farmers (67.5%) reported having a watering place in their fields or less than 100 meters from the fields; 13.63% of the farmers had a watering place situated between 100 and 500 meters from the fields. The survey revealed that water from 50% of the watering places was used for human consumption, 29.26% for diluting pesticides, and 26.96% for animal consumption.

### Types of ailments affecting farmers

**Figure 3:**
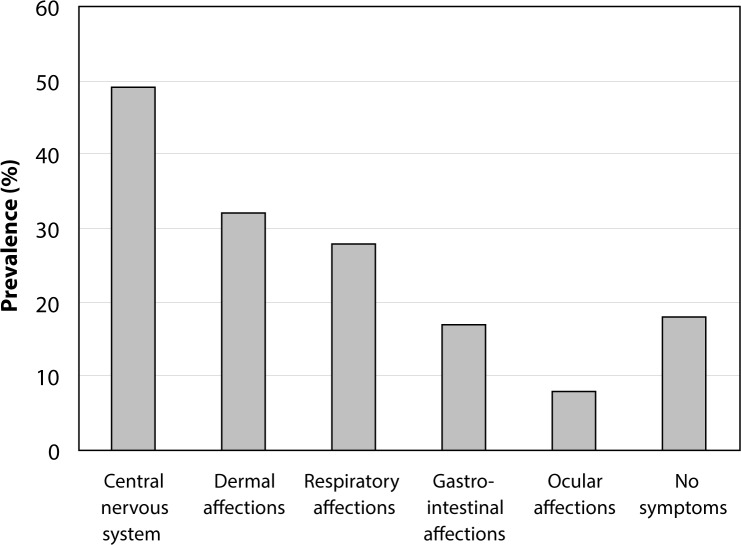
Distribution of farmers according to the type of ailments (reported by them) during or just after pesticide application in the fields.

**Figure 4:**
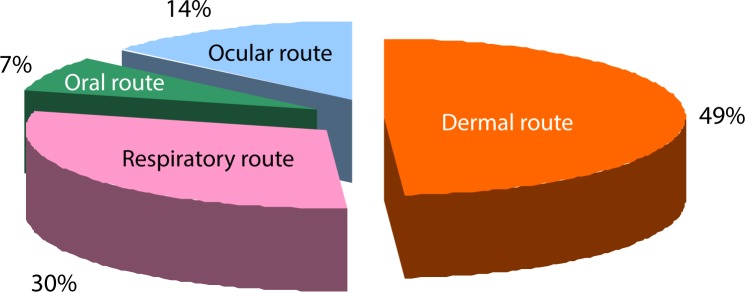
Exposure routes to pesticides reported by surveyed farmers.

Figure 3 shows the distribution of the different types of ailments affecting farmers during or just after pesticide application. The majority of the surveyed farmers (82.66%) reported having experienced, at least on one occasion, a feeling of ill-health during or just after pesticide applications. The exposure routes were dermal, respiratory, ocular and oral (Figure 4).

### Management of poisoning incidents by farmers

Table 2 summarizes the farmers’ attitude when poisoning incident would occur.

**Table 2:** Farmers’ attitudes when intoxication incident would occur.

Attitudes	Number	Percentages
Drinking milk	54	8.32
Drinking tamarind juice	15	2.31
Drinking lemon juice	13	2.00
Drinking sour juice	1	0.15
Drinking juice	2	0.31
Drinking coffee	2	0.31
Taking acetaminophen	1	0.15
Ingest charcoal and vomit	1	0.15
Go to healthcare center (CSPS)	25	3.85
Get rid of	7	1.08
Rub herself/himself with lemon leaves	20	3.08
Rub herself/himself with sorrel leaves	1	0.15
Rub herself/himself with vines	1	0.15
Apply ointment	1	0.15
Apply shea-butter	43	6.62
Wash with soap	540	83.20
Wash with potash soap	8	1.23
Wash with warm water	1	0.15
Wash with salted water	1	0.15
Suck sugar	1	0.15
No answer	8	1.23

### Poisoning data

**Figure 5:**
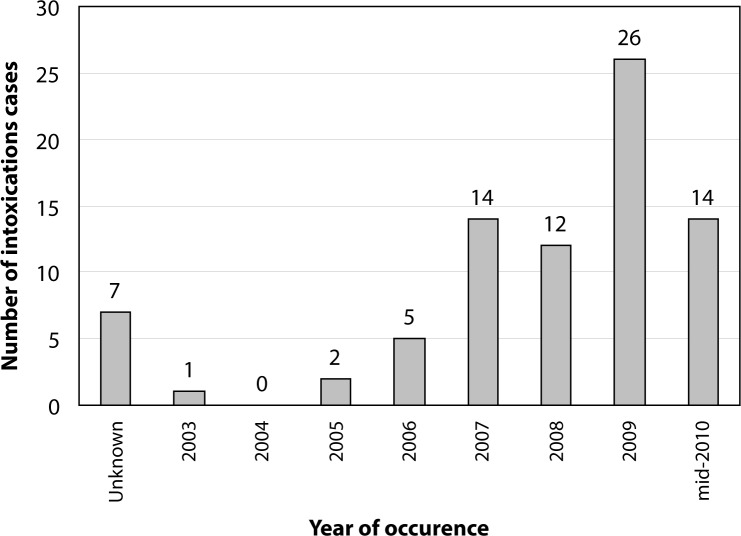
Distribution of the number of intoxication cases according to the year of occurrence.

A total of 42 healthcare centers were covered by the study, of which 40 health and social advancement centers and two health centers with surgical facilities (CMA). About 922 cases of pesticide poisoning (without detailed information) were reported. Pesticide poisoning cases reported with brief information included intoxication cases for which basic information is available. The information provided is related to the identity of the injured person (sex and age), the incident circumstance and its outcome. A total of 81 recorded poisoning cases fell into this category. The majority of victims were women (70.37%). The largest proportion of victims were adults (>19 years old) (54.33%), 19.75% were children (<14 years old), and 17.28% adolescents (14–19 years old). In 8.84% of the cases, age could not be identified. The majority of poisoning cases (53%) were due to unintentional ingestion of pesticides. It was reported that 28% of the cases were intentional (suicide) and 19% of the cases occurred while using pesticides in fields. As shown in Figure 5, the number of poisoning cases increased annually. The majority of victims, *i.e.* 80.25%, recovered whereas in 10% of cases poisoning was fatal. In 9.75% of cases, the outcome was unknown. Out of the 42 surveyed health officers, 20 (47.62%) declared not having much knowledge about pesticides, while 22 (52.37%) knew some facts about pesticides.

## Discussion

Certain behaviors and practices were identified to predispose to pesticide exposure and illness. The majority of the farmers using pesticides were relatively young (mean age 39.58 years). However, some were old, *i.e.* more than 50 years old (15.3%). This raises some concerns as it is known that the functional capacity of human vital organs, such as kidneys, decreases with age. Consequently, old age contributes to increase health risks related to the exposure of pesticides (Klaasen, 2007).

The large number of pesticides (153 products) used by farmers (which were often banned) could be factors contributing to health risks of pesticides (Mansour, 2004). Farmers usually combined insecticides of different classes in a single spray. Overall the level of education of the surveyed farmers was low (more than 60% of them are illiterates). They cannot read labels and follow recommended instructions for the proper use of pesticides. This fact does hinder the implementation of a scheme aimed at reducing health risks. However, farmers who have acquired literacy in the indigenous language can constitute an asset for the community. As a matter of fact, training programs on the management and proper use of pesticides can be designed and provided in the local language. Such programs could initially target a restricted number of individuals who will eventually be requested to take over training among the other members of the community.

The study showed that the extent of the farmers’ experience related to the use of pesticides varied considerably. About 54% of the farmers had between 10 and 30 years’ experience. This is very significant and indicates chronic exposure among these farmers (Konradsen, 2007). Contrary to the idea that experience can be an asset, we found that pesticide operators with the longest experience did not necessarily give the best example (Ouédraogo *et al.*, 2009). They were applying pesticides without personal protective equipments on the pretense that there were no risks in handling pesticides.

The conclusion drawn on pesticide management practices among farmers is that the careless habit of storing pesticides at home severely exposes family members to risks in terms of health, while discharging them into the environment or burying them inevitably leads to environmental contamination.

Pesticide application equipments used by the farmers were portable equipments which are manually operated. This situation also predisposed farmers to pesticide exposure. In India, it was found that tractor mounted techniques were only for big farms; the most commonly used equipment was hand-carried lever operated knapsack sprayer, which is not a very well designed mounted technique (Abhilash & Singh, 2009).

The scarce use of personal protective equipment and the tendency to have only partial protection inevitably leads to high exposure risks among pesticide applicators (Figure 2). Protection was usually incomplete, which outlines the different set of personal protective equipment worn by farmers during pesticide applications. Less than 1% of the farmers (0.93%) had full protection. The vicinity of watering sources to fields increases the risks of water contamination by pesticides released through different mediums.

Pesticides belonging to the WHO class I_b_ are highly hazardous and can be used only by certified and trained applicators and under close supervision. The use of such products should be strictly forbidden to farmers who have no training, who do not have appropriate personal protective equipment and who tend to underestimate pesticide-related hazards (WHO, 2004). Pesticides of Class II are considered as moderately hazardous and their use is restricted to trained applicators under close supervision who strictly comply with recommended precautionary measures. Some pesticides of WHO Class III were used; they are rated as slightly hazardous and can be used by trained applicators who comply with recommended precautionary measures. Well-trained farmers who would comply with recommended patterns of use and safety requirements should be able to handle these products with no major risk of intoxication. Pesticides of WHO class IV do not present acute hazards under normal use (WHO, 2004). Complying both with restrictions of use and precautionary measures is a way for pesticide applicators to ensure their safety.

Most farmers (82.66%) complained of discomfort during or just after pesticide applications while 17.34% of them never felt anything. Ailments affecting the central nervous system (experienced by 48.92% of farmers) were most reported by the farmers. As a matter of fact, exposure to insecticides is known to have severe adverse effects on the nervous system (Multinigner, 2005; Toe *et al.*, 2012).

As shown in Table 2, a large proportion of farmers had recourse to traditional medicine when intoxication incident would occur. This is not surprising as it is known that 80% of the populations in developing countries use medicinal plants to cure themselves (OMS, 2002). Only 3.08% of farmers would go to healthcare service centers.

The majority of the acute-poisoned patients were females and adults; this could be explained by the high prevalence of illiteracy among females in developing countries. Moreover, adults have free access to pesticides in rural areas. In fact, like in other developing countries, anyone is allowed to buy, handle and apply toxic agricultural chemicals without any necessary safety procedures (Lee & Cha, 2009). Thus majority of cases of pesticide poisoning cases were accidental (53%). The lethality due to pesticides poisoning was relatively high (about 10%); this could be explained by the inappropriate first aid attitude and the delay in admittance to healthcare centers.

## Conclusion

Particular socio-demographic factors, such as female sex, elderly age, and low education were related to increased risk of pesticides. Some attitudes and practices of farmers were also identified to predispose to agricultural pesticide exposure and illness in Burkina Faso. The management of agricultural pesticides in Burkina Faso was complicated by the number of different classes of pesticides which are highly or moderately toxic. Pesticide poisonings were relatively frequent. The most important policy change to reduce mortality from acute pesticide poisoning would be to phase out the most toxic chemicals, namely the WHO class I and II pesticides, and substitute them with less toxic groups of pesticides. Moreover, agricultural policies must reduce the use of pesticides to the lowest level feasible. Actions are needed to reduce pesticide poisoning as a global public health problem and to improve management of pesticide poisoning. To this purpose, advanced investigations should be carried out over a longer period of time to complement the present pilot study.
